# Prevalence and associated factors of electrocardiographic left ventricular hypertrophy in a rural community, central Thailand

**DOI:** 10.1038/s41598-021-86693-2

**Published:** 2021-03-29

**Authors:** Patipan Viwatrangkul, Sakda Lawanwisut, Pondfah Leekhaphan, Tatchamon Prasart-intara, Pathomphon Phiensuparp, Sirapat Prakiatpongsa, Promnavaporn Amnaj, Vichaya Phoominart, Krittanan Chanyou, Peeratuth Jiratrakan, Pisit Klumnaimueang, Nattapat Pipitdaecha, Rawin Panchamawat, Pannathorn Tangkongpanich, Mathirut Mungthin, Ram Rangsin, Boonsub Sakboonyarat

**Affiliations:** 1 Phramongkutklao College of Medicine, Bangkok, 10400 Thailand; 2 Division of Cardiology, Department of Medicine, Phramongkutklao College of Medicine, Bangkok, 10400 Thailand; 3 Department of Pharmacology, Phramongkutklao College of Medicine, Bangkok, 10400 Thailand; 4 Department of Military and Community Medicine, Phramongkutklao College of Medicine, Bangkok, 10400 Thailand

**Keywords:** Epidemiology, Population screening, Risk factors

## Abstract

Left ventricular hypertrophy (LVH) is considered a cardiac condition with life-threatening complications. Detected LVH is a strong predictor of cardiovascular diseases and death. This condition is normally diagnosed at offices. We aimed to determine the prevalence and associated factors of electrocardiographic-LVH (ECG-LVH) among adults in a Thai rural community. A cross-sectional study was conducted in Na-Yao rural community of Thailand in 2020. A total of 638 individuals aged ≥ 20 years were interviewed using standardized structured questionnaires related to demographic information, risk behaviors, comorbidities and anthropometric measurements. LVH was determined by Sokolov-Lyon and Cornell criteria based on the collected electrocardiograms. The prevalence of ECG-LVH among adults was 6.6%. The factors independently associated with ECG-LVH were being male (AORs 2.04, 95% CI 1.05–3.98), history of diabetes mellitus (AORs 1.01, 95% CI 1.01–1.02), and hypertensive crisis ≥ 180/110 mmHg (AORs 7.24, 95% CI 1.31–39.92). However, resting heart rate was negatively associated with ECG-LVH (*p* < 0.05). Our data emphasized that LVH was one of the significant health problems among adults in a rural community. This condition could lead to severe complications. Thus, effective detection and public health interventions should be provided at the community level.

## Introduction

Left ventricular hypertrophy (LVH) is considered a cardiac condition secondary to both structural and functional adaptation of the heart leading to many life-threatening complications like myocardial ischemia, left ventricular (LV) dysfunction, heart failure and even sudden cardiac death^1–4^. Several studies reported that the potential risk factors of LVH included being male^5,6^, high blood pressure^7,8^, insulin resistance^9^ and obesity^10^. Detecting and monitoring LVH, a strong predictor of cardiovascular diseases and death^11^, is crucial. Regression of LVH could reduce the risk of adverse outcomes^12^.

Approximately one half of the Thai population resides in rural areas where the characteristics of healthcare infrastructure and providers differ from those of urban settings especially in remote rural areas. In Thailand, a related study among adults in an urban setting in 2000 reported a prevalence of LVH at approximately 13%^13^. However, only limited information is available on the prevalence of and factors potentially responsible for LVH among adults in remote rural communities. The required information is essential to focus on preventing problems. Even though echocardiography is a gold standard to detect LVH, the technology is unavailable to use in those remote areas and also requires more specialists to complete the process. However, ECG criteria for LVH have been illustrated as strong independent predictors of cardiovascular morbidity and mortality in the general population^3,14^. Thus, this study aimed to determine the prevalence and associated factors of electrocardiographic-LVH (ECG-LVH) among adults in a remote rural community in central Thailand.

## Methods

### Study designs and subjects

The study was conducted in a remote rural area, 150 km from Bangkok: Na-Ngam community at the border area of Chachoengsao Province, central Thailand. The remote rural community houses approximately 3500 adults. A cross-sectional survey was conducted between January and February 2020. Information on people in the rural area was retrieved from the National Population Registry to determine the sampling frame. The registered population was selected using a random sampling which proportionated about 20% of population. The individuals residing in the area during the study were included. The inclusion criteria comprised adults aged at least 20 years old. Subjects were excluded from the study if they were pregnant (or suspected) or could not answer the questionnaires.

### Data collection

The investigators provided information sheets and informed subjects of the objectives and methods of the study including the interview questionnaire and ECG test. Written informed consent was obtained before conducting the study. When subjects could not read the information sheet, the investigators would read and describe the information to them. Additionally, subjects could use their fingerprint on the consent form to confirm their agreement. The duration of the questionnaire process, ECG testing and anthropometric measurement was approximately 30 min. Face-to-face interviews were performed using standardized questionnaires provided by well-trained interviewers. The questionnaire, used to identify the associated factors of ECG-LVH among adults in a rural community, covered information on demographic characteristics and self-reported comorbidity including history of hypertension (HT) and history of diabetes mellitus (DM), smoking and alcohol consumption. Smoking status was divided in three categories including current smoker, exsmoker and never^15^.

### Measurements

Body weight and height were measured using body composition monitor (OMRON model HBF-212, Kyoto, Japan) and stadiometer (DETECTO, St. Webb City, MO, USA), respectively. Body mass index (BMI) was categorized according to the Asia–Pacific BMI classifications, i.e., < 18.5 kg/m^2^, 18.5 to 22.9 kg/m^2^, 23.0 to 24.9 kg/m^2^, 25.0 to 29.9 kg/m^2^ and ≥ 30 kg/m^2^^16^. The participants stood on the floor with Frankfort horizontal plane^17^, then neck circumference was measured at midway between the midcervical spine and midanterior neck to within 1 mm, with a plastic tape^18^. Neck circumference was categorized in two groups, i.e., ≥ 35.75 cm (men), ≥ 32.75 cm (women), < 35.75 cm (men) and < 32.75 cm (women)^19^. Blood pressure was measured using an automatic blood pressure monitor (OMRON, HEM-7120, Kyoto, Japan) by an operator trained in standardized technique following the 2019 Thai guidelines on the treatment of HT^20^. The participants were advised to avoid caffeine and smoking for at least 30 min before the measurement was performed. The participants were instructed to be stationary at least 5 min in a chair, with feet on the floor and arms supported at heart level. During the measurement, talking was prohibited. Two measurements were taken, and the average was recorded.

Procedure for recording a standard 12-lead electrocardiography was conducted by well-trained investigators using a Digital 3-channel Color Electrocardiograph (Model: EKG-903A3, Beijing, PR China). The participants were asked to lie down in a comfortable position in a private partition room. The head was well-supported, the back rested on the bed and pillows. The inner aspects of the participants’ wrists were placed close to, but not touching, their waist. The investigator applied the limb leads at both wrists and ankles of the participants, then applied the chest leads; V1 to V6. The calibration signal on the ECG machine was checked to ensure standardization as a paper speed of 25 mm/s and ECG size 1 mV/10 mm deflection^21^. The participants were asked to lie still and to breathe normally during the measurement. The 12-lead ECG was recorded and correctly labeled within the identification number.

ECG-LVH was defined as a Sokolow-Lyon voltage (SV1 + RV5/6) > 35 mm^22^, and the Cornell voltage criterion-based LVH was defined as R in aVL + SV3 ≥ 28 mm for men S in V3 + R in aVL > 20 mm for women^23,24^. The ECG was reviewed, interpreted, and results were confirmed by cardiologists of Phramongkutklao Hospital in Bangkok.

The participants received the results, descriptions of their ECG test and the recommendation of self-health management. The participants presenting ECG-LVH and other abnormal ECG would receive standard care under their healthcare coverage scheme. The list of participants with ECG-LVH and other abnormal ECG patterns was registered in the health database at the Health Promoting Hospital, the primary care unit in Na-Yao rural community.

### Ethics consideration

This study was reviewed and approved by the Institutional Review Board, Royal Thai Army Medical Department, approval number R155q/62. The participants consented in agreement with the WMA Declaration of Helsinki–Ethical principles for medical research involving human subjects.

### Statistical analysis

Demographic characteristics were determined using descriptive statistics. Prevalence of ECG-LVH was determined using descriptive statistics and reported as a percentage with 95% confidence interval (CI). Binary logistic regression analysis was used to determine the associated factors of ECG-LVH, and multicollinearity was tested. Multivariate logistic regression analysis was performed; then adjusted odds ratio (AOR) was presented with corresponding 95% CI. A *p*-value less than 0.05 was considered statistically significant. Data were analyzed using StataCorp. 2019, Stata Statistical Software: Release 16. College Station, TX: StataCorp LLC.

## Results

### Characteristics of study participants

A total of 638 adults residing in a rural community were enrolled in the present study. Of all participants, 198 (31.1%) were male. The average age of the participants was 56.9 ± 13.0 years. Most participants obtained their highest education level from primary school accounting for 69.3%. In all, 560 (87.8%) applied the universal health coverage scheme. In terms of comorbidities, 37.9 and 11.1% of participants reported having a history of HT and DM, respectively. Average BMI among all study participants was 25.0 ± 4.4 kg/m^2^. Demographics of the study participants are presented in Table 1.

**Table 1:** Demographic characteristics of participants (n = 638).

Characteristics	n (%)
**Gender**	
Male	198 (31.0)
Female	440 (69.0)
**Age (years)**	
Mean ± SD	56.9 ± 13.0
20–29	20 (3.1)
30–39	37 (5.8)
40–49	125 (19.6)
50–59	188 (29.5)
60–69	153 (24.0)
≥ 70	115 (18.0)
**Marital status**	
Single	41 (6.4)
Married	485 (76.0)
Widow	86 (13.5)
Divorced	26 (4.1)
**Education level**	
Less than primary school	69 (10.8)
Primary school	442 (69.3)
Secondary school	45 (7.1)
High school	53 (8.3)
Vocational	10 (1.6)
Bachelor or higher	19 (3.0)
**Scheme**	
Universal health coverage	560 (87.8)
Government officer scheme	47 (7.4)
Social security scheme	25 (3.9)
Others	6 (0.9)
**Hypertension**	
No	396 (62.1)
Yes	242 (37.9)
**Diabetes mellitus**	
No	567 (88.9)
Yes	71 (11.1)
**Blood pressure (mmHg)**	
SBP mean ± SD	126.2 ± 16.1
DBP mean ± SD	76.3 ± 10.6
**Body mass index (kg/m**^**2**^**)**	
mean ± SD	25.0 ± 4.4
< 18.5	35 (5.5)
18.5–22.9	172 (27.0)
23.0–24.9	119 (18.7)
25.0–29.9	236 (37.0)
≥ 30.0	76 (11.9)

### Prevalence of ECG-LVH among adults in a Thai rural community

A total of 42 adults (6.6%, 95% CI 4.7% to 8.5%) were identified to have ECG-LVH. The prevalence of ECG-LVH among males was 10.7% (95% CI 6.3–15.0%) while the prevalence was 4.8% (95% CI 2.8–6.8%) among females. The prevalence of ECG-LVH was 7.0% (95% CI 3.8 to 10.3%) and 11.4% (95% CI 3.7 to 19.1%) among adults reporting a history of HT and DM, respectively.

### Factors associated with ECG-LVH among adults in a Thai rural community

Table 2 shows the univariate logistic regression analysis for factors associated with ECG-LVH among adults in this community. After adjusting for potential confounders, factors independently associated with ECG-LVH were identified and presented in Table 3. Male participants were associated with ECG-LVH (AOR 2.0, 95% CI 1.1 to 4.0). Participants reporting a history of DM were associated with ECG-LVH (AOR 1.01, 95% CI 1.01 to 1.02). Our findings indicated that adults with high blood pressure level revealing hypertensive crisis (SBP ≥ 180 mmHg or DBP ≥ 110 mmHg) were associated with ECG-LVH (AOR 7.2, 95% CI 1.3 to 39.9). A dose response relationship between resting heart rate (HR) and negative association with ECG-LVH was identified (*p* < 0.05). An increase in neck circumference tended to relate to ECG-LVH; however, without association (AOR 1.6, 95% CI 0.8–3.2).

**Table 2:** Univariate analysis factors associated with ECG-LVH among adults in a rural community.

Factors	ECG-LVH		Crude odds ratio	95% CI	*p*-value
	Yes	No			
	n (%)	n (%)			
**Gender**					
Female	21 (4.8)	419 (95.2)	1		
Male	21 (10.6)	177 (89.4)	2.37	1.26–4.44	0.007
**Age, mean ± SD, years**	58.1 ± 11.7	56.8 ± 13.1	1.01	0.98–1.03	0.530
< 50	14 (7.7)	168 (92.3)	1		
50–69	19 (5.6)	322 (94.4)	0.71	0.35–1.45	0.344
≥ 70	9 (7.8)	106 (92.2)	1.02	0.43–2.44	0.966
**Smoking**					
Never	29 (5.8)	469 (94.2)	1		
Current smoker	5 (6.6)	71 (93.4)	1.14	0.43–3.04	0.795
Ex-smoker	8 (12.5)	56 (87.5)	2.31	1.01–5.30	0.048
**Alcohol drinking**					
Never	12 (4.7)	242 (95.3)	1		
Current drinker	22 (8.6)	233 (91.4)	1.90	0.92–3.94	0.082
Ex-drinker	8 (6.2)	121 (93.8)	1.33	0.53–3.35	0.540
**History of diabetes mellitus**					
No	34 (6.0)	533 (94.0)	1		
Yes	8 (11.3)	63 (88.7)	1.99	0.88–4.49	0.097
**History of hypertension**					
No	25 (6.3)	371 (93.7)	1		
Yes	17 (7.0)	225 (93.0)	1.12	0.59–2.12	0.725
**Blood pressure, mean ± SD, mmHg**					
Systolic	132.4 ± 19.6	125.8 ± 15.8	1.02	1.01–1.04	0.012
Diastolic	75.4 ± 14.6	76.3 ± 10.3	0.99	0.96–1.02	0.570
**Hypertensive crisis**					
SBP < 180 mmHg and DBP < 110 mmHg	40 (6.3)	590 (93.7)	1		
SBP ≥ 180 mmHg or DBP ≥ 110 mmHg	2 (25.0)	6 (75.0)	4.92	0.96–25.15	0.056
**Heart rate, mean ± SD, bpm**	72.2 ± 13.3	79.1 ± 11.9	0.95	0.92–0.98	< 0.001
50–59	7 (24.1)	22 (75.9)	1		
60–69	11 (8.7)	116 (91.3)	0.42	0.13–1.32	0.137
70–79	11 (5.8)	178 (94.2)	0.27	0.09–0.86	0.026
80–89	8 (4.4)	175 (95.6)	0.20	0.06–0.67	0.009
≥ 90	5 (4.5)	105 (95.5)	0.21	0.06–0.79	0.020
**Body mass index, mean ± SD, kg/m**^**2**^	25.2 ± 5.3	25.0 ± 4.3	1.01	0.94–1.09	0.728
< 18.50	4 (11.4)	31 (88.6)	1		
18.5–22.99	10 (5.8)	162 (94.2)	2.09	0.62–7.09	0.237
23.0–24.99	6 (5.0)	113 (95.0)	0.86	0.30–2.43	0.777
25.00–29.99	15 (6.4)	221 (93.6)	1.10	0.48–2.51	0.822
≥ 30.00	7 (9.2)	69 (90.8)	1.64	0.60–4.50	0.333
**Waist circumference, mean ± SD, cm**	86.9 ± 10.9	84.8 ± 11.1	1.02	0.99–1.05	0.245
< 90 in male, < 80 in female	16 (6.0)	252 (94.0)	1		
≥ 90 in male, ≥ 80 in female	26 (7.0)	344 (93.0)	1.19	0.63–2.27	0.596
**Neck circumference, mean ± SD, cm**	35.4 ± 3.4	34.4 ± 3.2	1.10	1.00–1.20	0.050
< 35.75 in male, < 32.75 in female	12 (4.7)	246 (95.4)	1		
≥ 35.75 in male, ≥ 32.75 in female	30 (7.9)	350 (92.1)	1.76	0.88–3.50	0.109

**Table 3:** Multivariate analysis factors associated with ECG-LVH among adults in a rural community.

Factors	ECG-LVH		Adjusted odds ratio	95% CI	*p*-value
	Yes	No			
	n (%)	n (%)			
**Gender**					
Female	21 (4.8)	419 (95.2)	1		
Male	21 (10.6)	177 (89.4)	2.04	1.05–3.98	0.035
**History of diabetes mellitus**					
No	34 (6.0)	533 (94.0)	1		
Yes	8 (11.3)	63 (88.7)	1.01	1.01–1.02	0.033
**Hypertensive crisis**					
SBP < 180 mmHg and DBP < 110 mmHg	40 (6.3)	590 (93.7)	1		
SBP ≥ 180 mmHg or DBP ≥ 110 mmHg	2 (25.0)	6 (75.0)	7.24	1.31–39.92	0.023
**Heart rate, bpm**					
50–59	7 (24.1)	22 (75.9)	1		
60–69	11 (8.7)	116 (91.3)	0.28	0.09–0.83	0.021
70–79	11 (5.8)	178 (94.2)	0.19	0.06–0.58	0.004
80–89	8 (4.4)	175 (95.6)	0.14	0.04–0.46	0.001
≥ 90	5 (4.5)	105 (95.5)	0.14	0.04–0.51	0.003
**Neck circumference, cm**					
< 35.75 in male, < 32.75 in female	12 (4.7)	246 (95.4)	1		
≥ 35.75 in male, ≥ 32.75 in female	30 (7.9)	350 (92.1)	1.55	0.76–3.15	0.232
*Multivariate analysis* Adjusted for Gender, History of DM, Hypertensive crisis, Heart rate and Neck circumference					

## Discussion

Our data demonstrated the prevalence of ECG-LVH among adults residing in a Thai rural community was 6.6%. ECG-LVH was significantly associated with being male, history of DM, hypertensive crisis (SBP ≥ 180 mmHg, DBP ≥ 110 mmHg) and resting HR. To our knowledge, this was the first report on the prevalence and risk factors of ECG-LVH in a general Thai populations residing in a rural community. The prevalence of ECG-LVH among participants in the present study was relatively low when compared with other reports in both rural and urban settings. A related study among Thai people aged ≥ 30 years in an urban area in 2000 reported that the prevalence of ECG-LVH was 13%^13^. However, the prevalence of ECG-LVH among adults in a rural farm setting in Rivers State of Nigeria was 16.4%^5^. These wide gaps from the present study may be related to the fact that African populations tended to have a high ventricular mass when compared with other ethnics^25^.

Our finding reported that the prevalence of ECG-LVH among males was significantly higher than that identified among females. This finding was compatible with other related studies^5–7,13^; however, other studies reported a higher prevalence of ECG-LVH among females^26^. Although being male was an associated risk factor for ECG-LVH, LVH was a greater risk factor for cardiac death among females than among males^27^. Strong evidence was observed that ECG-LVH was a risk predictor of cardiovascular events including coronary heart disease, stroke and sudden cardiac death^2,3,8,28–30^. When LVH continued to progress, the prevalence of patients with cardiovascular diseases and their complications were more likely to increase. Thus, early detection, prevention and treatment of modifiable cardiovascular risk factors in a general population residing in a community should be implemented as effective interventions.

We found that study participants reported a history of DM related to ECG-LVH. Similarly, the NOMAS cohort study among a multi-ethnic population in the US showed that DM was independently associated with increased risk of LVH^25^. One recent study in the UK demonstrated a relationship existed between cardiac steatosis and LV geometric remodeling among patients with DM; however, the causality of this relationship will need to be investigated in the future^4^. Furthermore, related studies using murine models reported that cardiac steatosis, where superfluous triglyceride accumulates in the myocyte, led to cardiomyopathy^31,32^.

Regarding related reports in a rural community^7,33^, we found that ECG-LVH was linked to high blood pressure level, especially SBP ≥ 180 mmHg or DBP ≥ 110 mmHg. The finding was consistent with the related study of Tangjatuporn et al. reporting that 20% of patients with uncontrolled blood pressure were identified as presenting ECG-LVH^7^. Our study revealed that adults with high blood pressure as hypertensive crisis had a strong risk associated with ECG-LVH (AOR 7.2, 95% CI 1.3 to 39.9). High blood pressure increases LV wall stress triggering both neurohumoral activation and mechanical stress pathways. When these processes are not prohibited, hypertensive heart disease including cardiac arrythmias, heat failure and coronary heart disease will occur^34,35^. Furthermore, we found that 6 (75%) of a total of 8 participants with hypertensive crisis were unaware of HT. Our data suggested that effective community interventions such as HT screening and modified risk factors of uncontrolled blood pressure among adults with HT should be provided in rural settings^19^.

Related evidence has demonstrated that resting HR is a predictor of cardiovascular events including death^36,37^. However, our study reported that resting HR was negatively associated with ECG-LVH, significantly. Similarly, related studies in Egypt and Greece reported that LVH was reversely related to HR^38,39^. Additionally, the finding from the OGHMA study in Japan reported that each 10 beats/min increase in resting HR was associated with reduced development of ECG-LVH, especially among males^40^. Nevertheless, the temporal relationship between resting HR and developing ECG-LVH in the present study could not be proved due to the cross-sectional design.

The study employed a cross-sectional survey which could make it difficult to establish a cause-and-effect relationship between associated factors and ECG-LVH. Another limitation was the small sample size in the study; thus, the association between the well-known risk factors such as obesity and outcome could not be presented. When the study was conducted, the young adults especially those aged less than 40 years were unavailable to participate because most people in this age group (20 to 39 years) worked in the other areas. Thus, the prevalence of ECG-LVH in this community may have been overestimated. However, social desirability bias might also have existed in the study due to face-to-face interview, although the interviewers were well-trained and used standardized surveys.

The study identified a few modifiable risk factors for ECG-LVH which would be advantageous implementing prevention strategies at the community level. Adults, especially those residing in rural areas should be targeted to increase health literacy and raise awareness about LVH, its complications and adjust their modifiable risk factors especially controlling blood pressure. Authorities in rural communities such as healthcare workers at health promoting hospitals should provide HT and DM screening to identify potential risk factors for LVH. Then adults with HT or DM should be invited to receive a continuous care under the universal health coverage scheme for blood pressure, glycemic control and LVH early detection. Our study may not be generalized to the whole country but may reflect the real experience of adults residing in rural communities in Thailand.

## Conclusion

Our data emphasized that ECG-LVH was a significant health issue among adults residing in a rural community in Thailand. Effective public health interventions, especially controlling blood pressure and raising awareness of HT status, should be provided in the community to reduce risks of ECG-LVH. The modifiable risk factors for ECG-LVH should be attenuated to inhibit the progression of cardiovascular events including coronary heart disease and cardiac mortality.

## Data Availability

The datasets generated during and/or analyzed during the current study are not publicly available because the data set contains sensitive identifying information. Because ethical restrictions have been placed, the data sets are available from the corresponding author on reasonable request.

## Abbreviations

ECG-LVH: Electrocardiographic-left ventricular hypertrophy
LV: Left ventricular
HT: Hypertension
DM: Diabetes mellitus
HR: Heart rate
SBP: Systolic blood pressure
DBP: Diastolic blood pressure
OR: Odds ratio
AOR: Adjusted odds ratio
95% CI: 95% Confidence interval
